# GFAP as a marker of astrocytic damage correlated with medication overuse in migraine

**DOI:** 10.1007/s12026-025-09674-x

**Published:** 2025-08-09

**Authors:** Sara Carta, Vanessa Chiodega, Riccardo Tiberi, Alessia Pasquali, Sergio Ferrari, Silvia Bozzetti, Federico Ranieri, Fabio Marchioretto, Sara Mariotto

**Affiliations:** 1 Department of Neurosciences, Biomedicine and Movement Sciences, Neurology Unit, University of Verona, Policlinico GB Rossi, P.Le LA Scuro 10, Verona, 37135 Italy; 2 Experimental and Clinical Medicine Department, Neurological Clinic, Marche Polytechnic University, Ancona, Italy; 3 Department of Neurology/Stroke Unit, San Maurizio Hospital, Bolzano, Italy; 4 Neurological Unit, IRCCS Sacro Cuore Don Calabria Hospital, Negrar Di Valpolicella, Verona Italy

**Keywords:** GFAP, Astrocytic damage, Medication overuse, Migraine

## Abstract

Different mechanisms are involved in migraine pathogenesis, including neurogenic inflammation, neurodegenerative processes, and a potential role of microglia. The aim of this study was to assess axonal and glial damage measuring serum levels of neurofilament light chain (NfL) and glial fibrillary acidic protein (GFAP) in migraine patients. Serum samples of 25 patients with episodic migraine (EM), 25 with chronic migraine (CM**)** diagnosed in accordance with the International Classification of Headache Disorders, 3rd edition (ICHD-3), and 50 age-matched healthy controls were prospectively collected. NfL and GFAP levels were assessed using ultrasensitive paramagnetic bead-based ELISA (SIMOA). Non-parametric tests were used for group comparison and 2-tailed Spearman analysis to assess correlations. GFAP levels were significantly increased in migraine patients (median 103.15 pg/mL [*IQR* 70.98–146.34] vs. 69.43 pg/mL [*IQR* 53.04–91.85], *p* < 0.001), particularly in those with medication overuse (106.08 [*IQR* 87.94–159.07] vs. 71.38 [*IQR* 54.16–135.06], *p* = 0.007), without difference between EM and CM (*p* = 0.985). Although NfL levels were not increased (*p* = 0.387), they were higher in patients with a long migraine course (rho 0.519, *p* < 0.001). Attack at sampling/days from last attack, migraine frequency/attack severity did not influence NfL or GFAP levels. Our findings demonstrate the occurrence of glial damage, particularly correlated with medication overuse, and the presence of axonal damage in the later disease stage, providing potential novel cues for the migraine pathogenesis.

## Background

Migraine is a chronic neurological disorder that affects over 1 billion people globally with a 1-year prevalence of up to 18% in the adult population [1]. Although historically being considered a benign disorder, migraine attacks can be severely disabling, impacting patients’ mental, physical, and social well-being [1–3].

Diagnosis is primarily based on clinical evaluation, through the application of the International Classification of Headache Disorders (ICHD-3) [4].

The pathophysiology of migraine is not completely understood, but neurogenic inflammation, microglia, and neurodegenerative processes might play a role [4–6]. Previous studies on animal models suggest that astrocytes are involved in cortical spreading depression, neuroinflammation, and central sensitization [5, 7, 8].

Recent investigations on blood biomarkers suggest the possible occurrence of underlying subtle neuronal and glial damage. Previous studies have explored the role of neuron-specific enolase (NSE) with contradictory results [9, 10]. Serum neurofilament light chain (NfL), a well characterized marker of axonal damage, has been demonstrated to be slighter increased in young patients with a long disease history (> 10 years) [11, 12]. Studies focusing on S100 calcium-binding protein B (S100B), which is expressed mainly in glial cells, led to conflicting results but overall reported more elevated concentration in migraineurs patients [10, 13, 14]. CX3CL1, a regulator of microglial activation, has been found to be elevated in the CSF of migraine patients, while CSF concentration of TREM2, another biomarker of microglia activation, was not increased [15].

The potential role of GFAP, a well-recognized biomarker of astrocytic damage, was recently investigated in migraine with diverging results [16–18].

The aim of this study is to evaluate serum levels of NfL and GFAP in patients with episodic migraine (EM) and chronic migraine (CM) in comparison with healthy age-matched controls to clarify the correlation between migraine and axonal/glial damage and to explore the utility of these biomarkers in the clinical practice.

## Methods

Patients with migraine were prospectively recruited from two dedicated Neurology units (AOUI and IRCCS Sacro Cuore Don Calabria Hospital, Verona, Italy) from August 2023 to July 2024. Age-matched healthy controls (HC) were also included for comparison.

The study was approved by the local ethic committees (Prog. 4066CESC and Prog. 19CET), and informed consent was obtained from all patients and controls.

Inclusion criteria were (a) either presence (for patients) or absence (for HC) of EM or CM diagnosed according to the ICHD-3 [19], (b) age at sampling between 18 and 50 years old, (c) exclusion of other headache disorders including hemiplegic migraine, cluster headache or secondary headache disorders other than medication-overuse headache, (d) absence of other neurological or psychiatric comorbidities, and (e) absence of systemic conditions possibly influencing biomarkers levels (including kidney dysfunctions).

The fulfillment of inclusion/exclusion criteria for migraine patients was verified by two neurologists specialized in headache (FM and FR). Healthy controls were volunteers recruited by neurologists (SC, RT, and AP).

Demographic and clinical data including age, sex, comorbidities, attack presence at sampling, days from last attack, migraine frequency (monthly migraine headache days), mean severity of attacks during the previous month (collected through visual analogue scale -VAS-), migraine course (years), and presence of aura before attacks were collected in a dedicated database. Treatment strategies including previous preventive treatments, actual preventive treatments, attack medications, and history of medication overuse in the previous 2 years, as defined in previous studies [20] were also analyzed.

Blood samples were obtained and centrifuged for 10 min at 800 g at room temperature. Serum was stored within 2 h at − 80 °C until sample analysis. Investigators blinded to clinical data measured serum NfL and GFAP levels using SIMOA two-plex kit in SR-X immunoassay analyzer, Simoa (Quanterix Corporation, Billerica, Massachusetts, USA), which runs ultrasensitive paramagnetic bead-based enzyme-linked immunosorbent assays. The analysis was performed at the Neuropathology and Neuroimmunology Laboratory, University of Verona, Italy, according to manufacturer’s instructions. Briefly, 25 μL of frozen samples and calibrator was equilibrated to room temperature and diluted with specific sample diluent. Calibrators, samples, detector, and beads were dispensed in each well, and plates were incubated at 30 °C with shaking 800 rpm for 30 min. After washing steps, 100 μL SBG was added to each well, and plates were incubated at 30 °C with shaking 800 rpm for 10 min. After washing steps, beads were resuspended twice at 1000 rpm for 1 min. A final washing step was performed, and plates were dried for 10 min before being transferred to the SR-X reader [21, 22].

Sample size (migraine patients vs. HC) estimation was based on NfL and GFAP levels previously reported, assuming a clinically meaningful difference of 20%, a significance level of 0.05, and a statistical power of 80% [23, 24].

Descriptive statistics were performed using median (interquartile ranges [*IQR*]) and percentages for categorical variables). Group comparisons (migraine patients vs. HC and EM vs. CM) were assessed using non-parametric tests (*χ*^2^ and Mann–Whitney test), as appropriate. Correlation analyses between biomarkers and relevant clinical features were performed using a 2-tailed Spearman analysis with a Bonferroni correction for multiple comparisons. Analyses were performed using IBM SPSS 25; *p* values < 0.05 were considered statistically significant.

## Results

A total of 50 patients with migraine and 50 HC were enrolled. Median age at sampling was 37 years old [*IQR* 33–41] in healthy controls and 39 years old [*IQR* 30–46] in migraine patients (*p* = 0.542). Female sex was more frequent in migraine patients (82% vs. 60%, *p* = 0.015). Among migraine patients, 25 (50%) had EM while 25 (50%) were affected by CM. A history of medication overuse was present in 30 (60%) of cases, more commonly in CM patients (*p* = 0.001). Detailed clinical information is reported in Table 1.

**Table 1:** Characteristics of patients with migraine

	Migraine patients (all) (*n* = 50)	Migraine patients with episodic migraine (*n* = 25)	Migraine patients with chronic migraine (*n* = 25)	*p*
Age, median [*IQR*]	39 [30–46]	36.5 [29–43.75]	43 [31.75–46.25]	0.189
Female, *n* (%)	41 (82)	20 (83.3)	21 (80.8)	0.814
Migraine frequency (monthly migraine headache days), median [*IQR*]	14.5 [5.3–20]	5 [2.25–10]	20 [18–25.75]	< 0.001
Migraine attacks at sampling, *n* (%)	15 (30)	5 (20)	10 (40)	0.095
Days from last attack, median [*IQR*]	2 [0–7] *n* = 34	7 [0–14]	0 [1–3]	**0.036**
Attack severity, VAS, median [*IQR*]	8 [7–9]	7.5 [6.5–8.75]	8 [7–9.63]	0.143
Presence of aura, *n* (%)	4 (8)	3 (11.54)	1 (3.85)	0.338
Migraine duration (years)	23.5 [15.75–29.25]	20 [13.5–29]	24.5 [17.25–29.25]	0.648
Medication overuse, ever, *n* (%)	30 (60)	9 (36)	21 (84)	**0.001**
Acute treatment, NSAID, *n* (%)	35 (50)	10 (40)	15 (60)	0.053
Acute treatment, triptans, *n* (%)	39 (78)	20 (80)	19 (76)	0.562
Preventive treatment at sampling, any (%)	28 (56%)	12 (48)	16(64)	0.242
Preventive treatment type				
Beta blockers	6 (12)	2 (8)	4 (16)	
Amitriptyline	8 (16)	0	8 (32)	
ASMs	22 (44)	8 (32)	14 (56)	
CGRP-mAbs	8 (16)	6 (24)	2 (8)	
Previous treatment, any	30 (60)	10 (40)	20 (80)	0.094
Serum NfL, pg/mL, median [*IQR*]	8.87 [7.04–12.96]	8.63 [7.37–12.86]	10.37 [6.61–13.3]	0.806
Serum GFAP, pg/mL, median [*IQR*]	103.15 [70.98–146.34]	105.97 [64.02–148.08]	98.81 [76.99–133.78]	0.985

*IQR* interquartile range, *VAS* visual analogue scale, *NSAID* nonsteroidal anti-inflammatory drug, *ASM* anti-seizure medication, *CGRP-mAbs* calcitonin gene-related (CGRP) peptide or the CGRP receptor monoclonal antibodies, *NfL* neurofilament light chain, *GFAP* glial fibrillary astrocytic protein. Significant results are marked in bold

**Fig. 1:**
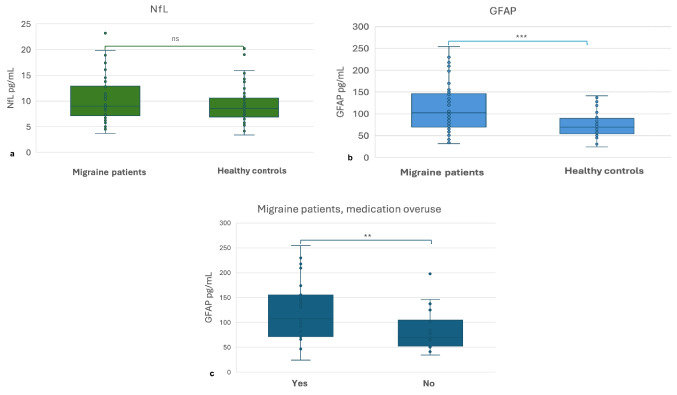
Serum levels of NfL and GFAP in migraine patients and healthy controls. **a** NfL levels are not significantly different in healthy controls and migraine patients (median 8.58 [*IQR* 6.74–11.04] vs. 8.87 pg/mL [*IQR* 7.04–12.96], *p* = 0.387). **b** *GFAP* levels are significantly increased in migraine patients, compared to healthy controls (median 69.43 pg/mL [*IQR* 53.04–91.85] vs. 103.15 pg/mL [*IQR* 70.98–146.34], *p* < 0.001). **c** GFAP levels are significantly increased in migraine patients with a history of medication overuse in comparison with patients without medication overuse (median 105.97 [*IQR* 86.87–162.64] vs. 71.38 [*IQR* 54.16–135.06], *p* = 0.007)

Serum NfL median levels did not significantly differ between HC and migraine patients (8.87 [*IQR* 7.04–12.96], vs. 8.58 pg/mL [*IQR* 6.74–11.04] *p* = 0.387) (Fig. 1).

GFAP levels are significantly higher in migraine patients (103.15 pg/mL [*IQR* 70.98–146.34] vs. 69.43 pg/mL [*IQR* 53.04–91.85], *p* < 0.001), without significant difference between EM and CM patients and between patients with and without an attack at sampling (*p* = 0.235) (Table 1). Of note, GFAP levels were significantly increased in patients with a history of medication overuse (106.08 [*IQR* 87.94–159.07] vs. 71.38 [*IQR* 54.16–135.06], *p* = 0.007) (Fig. 1). Of note, age at sampling (43 [*IQR* 32.3–46] vs. 33.5 [*IQR* 29–43.3] *p* = 0.134) and disease duration (22.5 [*IQR* 13.8–29] vs. 24 [16–26,27*p* = 0.858) did not significantly differ between patients with and without medication overuse. When comparing migraine patients without medication overuse and HC, NfL, and GFAP levels did not differ significantly (GFAP 71.38 pg/mL [*IQR* 54.86–108.08] vs. 69.43 pg/mL [*IQR* 53.04–8.99], *p* = 0.696*;* NfL 8.61 pg/mL *IQR* [7.08–10.88], *p* = 0.743)*.*

Correlation analysis between NfL, GFAP, age at sampling, migraine course, migraine frequency, days from last attack, and attack severity showed an association between NfL and age at sampling (rho 0.643, *p* < 0.001) and NfL and migraine course (rho 0.519, *p* < 0.001).

## Discussion

In this case–control study, we observed that (a) GFAP serum levels are significantly increased in migraine patients, particularly in those with medication overuse; (b) NfL levels are not increased in migraine patients; (c) attack at sampling/days from last attack and migraine frequency/attack severity do not influence NfL and GFAP levels; and (d) NfL but not GFAP levels are higher in patients with a long migraine course.

GFAP is a well-recognized biomarker of astrocytic damage. Its role as a marker of disease severity and disease activity has been demonstrated in several other neurological disorders, including traumatic brain injury, neurodegenerative diseases, and inflammatory disorders such as multiple sclerosis and neuromyelitis optica spectrum disorder [25, 26].

Astrocytes seem to be involved also in migraine pathogenesis, contributing to initiation and propagation of cortical spreading depression and actively participate in neuroinflammation and central sensitization [7, 8, 27]. Although findings from animal models may not always be directly translatable to human diseases, alterations in the immunoreactivity of GFAP-positive astrocytes have been reported in response to chronic migraine-associated pain [5]. Moreover, astrocyte dysfunction alters neuronal activity in the cingulate cortex and facilitates migraine-like cranial pain states in a mouse model of migraine [28]. Finally, a previous study detected an increase in GFAP values in the spinal trigeminal nucleus of mice that received an injection of calcine-gene related peptide in the trigeminal ganglion [29].

So far, only a few studies have investigated the potential role of GFAP as a biomarker in migraine with conflicting results [16–18]. Of note, medication overuse was not fully investigated before, and not all studies included patients under preventive treatment, therefore potentially excluding patients with a more severe disease. Our results, in agreement with the largest study recently published [16], indicate that migraine is associated with a low grade of astrocyte damage, reflecting astrocyte activation in pain modulation.

Astrocyte damage appears to be more prominent in patients with medication-overuse headache, a well-recognized risk factor for migraine progression [20]. Previous studies suggest that individuals with medication overuse headache may develop neuronal hyperexcitability due to insufficient acute pain relief, which can contribute to further central sensitization [30, 31]. The more pronounced elevation of GFAP levels observed in these patients may reflect a more severe condition and a deeper impairment in pain modulation systems, in which astrocytes are thought to play a critical role.

The absence of correlation between attack stage/frequency might be related to the kinetics of GFAP, which is not completely understood, and might reflect the chronic rather than acute relation of migraine with astrocytic involvement. Of note, when migraine severity was assessed through VAS, there was no association between VAS values and GFAP levels. However, VAS has the limitation of being a subjective measure [32] and probably not completely reliable, as most patients reported a score between 7/10 and 9/10.

Finally, our data confirm previous studies reporting slightly increased NfL values in patients with a long disease course, suggesting that axonal damage might occur in a later disease stage [12].

The present study has limitations, particularly related to the small number of patients with EM and CM enrolled. Although the low sample size prevents definitive conclusions, our data suggests the occurrence of astrocytic damage, particularly in patients with medication overuse, and the presence of axonal damage in late disease stage providing novel insights in migraine pathogenesis, give novel cues for the use of efficacious preventive treatment to avoid the accumulation of axonal and glial damage.

## Data Availability

Anonymized data not published within this article will be made available by request from any qualified investigator

## Abbreviations

ICHD: International Classification of Headache Disorders
GFAP: Glial fibrillary acidic protein
S100B: S100 calcium-binding protein B
NSE: Neuron-specific enolase
NfL: Neurofilament light chain
CX3CL1: C-X3-C motif chemokine ligand 1
TREM2: Triggering receptor expressed on myeloid cells-2
EM: Episodic migraine
CM: Chronic migraine
HC: Healthy controls
VAS: Visual analogue scale
*IQR*: Interquartile range
